# Editorial for the Special Issue on Recent Advances in Microwave Components and Devices

**DOI:** 10.3390/mi15020180

**Published:** 2024-01-25

**Authors:** Jingchen Wang, Wencheng Lai

**Affiliations:** 1 Department of Communications and Networking, School of Advanced Technology, Xi’an Jiaotong-Liverpool University, Suzhou 215123, China; 2 Department of Electronic Engineering, National Yunlin University of Science and Technology, Douliu 640301, Taiwan

Microwave components and devices are essential elements of many key communication, sensing, and monitoring systems [1,2,3,4]. Continuous advancements in microwave engineering capabilities are becoming increasingly important as our reliance on wireless connections and high-frequency electronics in the commercial, medical, and industrial domains grows [5,6]. This Special Issue provides examples of current and emerging advancements that will influence the upcoming generation of microwave components and systems. The articles in this Special Issue cover significant aspects of innovation throughout the field of microwave engineering, including current advances in the modeling, design, and fabrication of RF front-ends, filters, phase shifters, power amplifiers, and antennas, as well as an advanced microwave sensor as an alternative for detecting dimethyl sulfoxide (DMSO) in water and a programmable multi-functional metasurface for microwave beam shaping. These articles highlight both incremental and disruptive improvements that assist in consolidating microwave technology as a versatile backbone of progressing wireless systems and applications.

This Special Issue covers both the depth and breadth of our developing understanding of microwave engineering foundations, as well as their use in specific applications. We hope that it will be a valuable reference for microwave researchers and practitioners, as well as an inspiration for future work to address remaining issues in analysis, design, and implementation. By highlighting present microwave developments as well as predictions for the future, this compilation serves as both a snapshot of the current situation and a forewarning of microwaves’ critical role in future technological convergence.

The primary avenues for elevating communication speed include heightening modulation orders or applying wider channel bandwidths. However, given the finite spectral assets within microwave spans, utilizing elevated modulation schemas to optimize spectrum exploitation still falls short of realizing rates of tens of Gbps. Conventional millimeter-wave discrete modules are costly and suffer from considerable inter-component losses. Moreover, dissociated conceptions, disjointed implementations, and mechanical assemblies of millimeter-wave functional blocks now fail to suit next-generation mobile communication systems’ price, power, and integration needs. Thereby, investigating silicon-based, exceptionally integrated RF front-ends is vital for prospective millimeter-wave wireless communication frameworks. A 40–50 GHz transmitter (TX) front-end with several integrated calibration capabilities is proposed (Contribution 1), including a differential quadrature mixer, a power amplifier, and a detection mixer. It uses a quadrature mixer with amplitude and phase tuning to achieve over 30 dB of image rejection, avoiding post-calibration. A detection mixer provides feedback to calibrate and suppress local oscillator (LO) leakage by over 10 dB. The TX delivers over 13.5 dBm of output power and meets the error vector magnitude requirements of the IEEE 802.11aj standard. The quadrature mixer’s in-phase quadrature (IQ) signal generator enables >30 dBc image signal rejection and >30 dBc LO leakage suppression.

Filters are an indispensable element needing continuous advancement to support nearly all advancing communication and electronic systems through selectivity, speed, efficiency, and integration. As modern communication systems and electronics become more complex and operate at higher frequencies, such as 5G and beyond, pushing filter performance in multiple dimensions thus remains a priority area. A novel synthesis of a quasi-Chebyshev *N*th-order stub-loaded coupled line ultra-wide bandpass filter (UWB-BPF) is designed with higher electrical performance and a reasonably high fractional bandwidth of 82.6% for seventh-order UWB-BPF (Contribution 3), which can achieve increased selectivity and equal ripple response. In another study, an ultra-compact low-pass spoof surface plasmon polariton filter based on an interdigital structure is presented with efficient transmission in the 0–5.66 GHz passband, excellent out-of-band suppression (over 24 dB) in the 5.95–12 GHz stopband, and ultra-shape roll-off at 5.74 GHz (Contribution 6). A space mapping modeling approach integrating mesh distortion is formulated to coalesce the computational effectiveness of the coarse model with the meticulousness of the fine model, encased within a four-pole waveguide filter architecture (Contribution 4). This approach generates an accurate replica with superior training statistics while simultaneously optimizing the modeling promptness.

A phase shifter constitutes a pivotal mechanism within the radio frequency front-end architectural hierarchy. Its phase aberration and insertion attrition quantitative metrics are determined to control the precision of the phase regulation integrated into each distinct radio frequency traversal, thereby subsequently influencing the caliber of the beam steering and scanning functional vectors. Consequently, creating supreme-performance phase shifter circuitry remains imperative. To overcome certain challenges such as high insertion loss behavior within constrained frequency allotments, ardor integration in applications beyond 90°, and deficient bandwidth performance at considerable phase deviation, a novel switched network based on a reverse short-circuited coupled line is designed for constructing a broadband 180-degree phase shifter with dimensions of 0.67 × 0.46 mm^2^, realized via 0.15-micrometer GaAs pHEMT technology (Contribution 5). The proposed phase shifter has an insertion loss of less than 2 dB, a return loss of larger than 12 dB, a maximal phase error of less than 0.6, and a channel amplitude difference of under 0.1 dB within the frequency range of 10–20 GHz. Another study presents a novel 8–18 GHz 90 T-type phase shifter with an area of merely 0.53 × 0.86 mm^2^ fabricated using 0.15 µm GaAs pHEMT technology (Contribution 7). The phase error and insertion loss (IL) of the proposed phase shifter are less than ±1.5° and 2.6 dB at 8–18 GHz, respectively. The well-behaved amplitude and phase distortion metrics corroborate the proposed architecture’s suitability for broadband applications necessitating sustained reliability across an extensive operational bandwidth. Moreover, a power amplifier also plays a vital role in microwave systems. To achieve a compact size, a Ku-band broadband power amplifier microwave monolithic integrated circuit with an area of 3.3 × 1.2 mm^2^ and based on 0.15 µm GaAs pHEMT technology is proposed to achieve a high-power gain and high-power design (Contribution 9). The peak power of the proposed power amplifier is 30.8 dBm at 16 GHz, and the output power is larger than 30 dBm, with an efficiency of more than 30% at 15–17.5 GHz. The fractional bandwidth of the 3 dB output power is 30%.

To fulfill the requirement for a compact structure with stable radiation performance, a single-port planar dual-broadband antenna with a dual-feed structure is proposed to accommodate the mobile communication bands covering 0.64–1 GHz and 1.59–2.82 GHz with a gain of 1 and 2.2 dB, respectively, for both bands (Contribution 10). For wearable applications, a textile bandwidth-enhanced polarization-reconfigurable half-mode substrate-integrated cavity antenna is presented (Contribution 11). A slot is cut out from the patch of a basic textile HMSIC antenna to excite two close resonances to form a wide bandwidth in the range of 2.29–2.63 GHz. By adjusting the position of the snap buttons, the RHCP and LP radiations are realized at 2.42 GHz with buttons OFF and ON, respectively.

Additionally, a multi-functional metasurface with an array of programmable unit cells for different microwave beam-shaping applications is demonstrated, which enables its usage in microwave imaging, information transmission, and sensing applications (Contribution 2). The phase delay of the microwave reflected by the metasurface can be switched at 6.2 GHz. Furthermore, a microwave sensor is proposed to accurately measure the concentration of DMSO in water/DMSO binary mixtures (Contribution 8). The proposed sensor utilizes microwave frequency measurements to determine the DMSO concentration, resulting in a non-invasive and efficient method of analyzing DMSO solutions.

We hope that this Special Issue on microwave components and devices will provide readers with a useful overview of current state-of-the-art developments within these rapidly expanding fields of study, as well as an introduction to several of the most cutting-edge techniques developed in this field. We would like to express our appreciation to all authors for contributing to this Special Issue. Moreover, we are grateful to the reviewers for dedicating their time as well as maintaining the standard of the publications included in this Special Issue. Finally, we are grateful to the staff of the Editorial Office of Micromachines, particularly Mr. Lebron Tu, for their invaluable assistance.
